# Acrocyanosis: The Least Known Acrosyndrome Revisited With a Dermatologic Perspective

**DOI:** 10.1155/drp/2904301

**Published:** 2025-01-16

**Authors:** Deniz Demircioğlu, Emel Öztürk Durmaz

**Affiliations:** Department of Dermatology, Acıbadem University School of Medicine, İstanbul, Turkey

**Keywords:** acrocyanosis, acrosyndromes, BASCULE syndrome, Bier spots, chilblain-like lesions, chilblains, coronavirus, COVID fingers, COVID toes, raynaud's disease

## Abstract

**Background:** Acrocyanosis is a functional peripheral vascular disorder, currently categorized under the canopy of acrosyndromes, i.e., a group of clinically similar and significantly overlapping vascular disorders involving the acral skin. The disorder might be primary or secondary, depending on the cause. Recently, there has been a remarkable surge in acrocyanosis prevalence along with the COVID-19 pandemic. Both COVID-19 infection and vaccines for COVID-19 have been affixed to the list of disorders instigating acrocyanosis.

**Objectives:** The goal of this narrative review was to evaluate the existing literature, project acrocyanosis from the viewpoint of dermatologists in the face of the COVID-19 pandemic, and assess the need for targeted research, education, and/or clinical practice.

**Methods:** An English literature search was conducted using PubMed and Google. All abstracts on acrocyanosis, irrespective of the article type and publication date, were retrieved and reviewed and those most relevant for the focus of this article were selected and summarized.

**Discussion/Results:** A narrative review was carried out. There is paucity of randomized, double-blind, placebo-controlled studies on acrocyanosis in the English literature, implicating the need for targeted research. Pertinent information still relies on anecdotal observations, case reports, case series, or scarce reviews, which are dated rather old and published in vascular-oriented journals. The scarcity of published literature on acrocyanosis in dermatology-oriented journals points to the necessity of professional education and improvement of clinical diagnostic skills for dermatologists.

**Conclusions:** Although acrocyanosis is the least known and the least studied acrosyndrome, it is increasingly more commonly confronted in the COVID-19 era. The diagnosis still largely relies on clinical findings. Accordingly, it has become a growing necessity for a dermatologist to remain updated on this peculiar disorder and be able to differentiate acrocyanosis from clinically similar cold-induced or cold-exacerbated acrosyndromes. Acrocyanosis is still misdiagnosed, underdiagnosed, underreported, and undertreated by the dermatology community.

## 1. Introduction, Definition, and History

The term acrocyanosis (AC), initially coined by Crocq in 1896, is derived from the Latin words kyanos (blue) and akron (extremity) [1, 2]. Currently, it is defined as a functional peripheral vascular disorder, characterized by painless, persistent, symmetrical, bluish to livid, cyanotic discoloration of acral skin, aggravated by cold exposure [3–11]. Despite an upward escalation in its prevalence because of the COVID-19 pandemic, what we know about this peculiar disorder remains as the tip of the iceberg in 21^st^ century.

The aim of the present article was to provide an updated narrative review on AC from a dermatological point of view, delineate its clinical imitators, outline current and future perspectives, and assess the need for additional targeted research, education, and/or clinical practice.

## 2. Methods

We conducted a PubMed and Google search of English-language articles using the following predetermined key terms: acrocyanosis, acrosyndromes, COVID fingers, COVID toes, chilblains, and chilblain-like lesions. We reviewed all the abstracts, irrespective of the article type and publication date, and retrieved and summarized those most relevant for the scope of this review.

### 2.1. Epidemiology

Although the exact prevalence remains obscure, AC is an extremely rare condition compared to other acrosyndromes [1, 7, 12, 13]. Nevertheless, the prevalence has started to rise in the face of the COVID-19 pandemic [14, 15]. The disorder is more common in children, adolescents, and adults younger than 25–30 years and shows a predilection for the female gender [1, 4, 12, 16].

### 2.2. Classification

AC is classified as primary (idiopathic/essential) or secondary, depending on the cause [1, 4, 5, 7]. Primary AC is a benign disorder without an apparent or demonstrable previous disease [4–7, 9, 13, 17]. Familial cases hint at genetical susceptibility, and preferential affliction of young females during the reproductive period points to a contributing role of hormonal factors [9, 16]. Secondary AC, without a gender or age predilection, may arise from occult or overt pathological conditions and sometimes it may represent the initial manifestation of a primary underlying disease [9, 17, 18]. An exhausted list of associated, prompting, or underlying disorders exists (Table 1). Resolution of AC with treatment of the primary condition, or with disappearance of the presumed causative factor, or with withdrawal of the perpetrator agent altogether alludes to its secondary nature [9].

### 2.3. Pathophysiology

Although the precise pathophysiologic mechanism is unclear, AC is ascribed to reduced oxyhemoglobin within the dermal and hypodermal blood vessels [1, 4–11]. Tissue hypoxia may stem either from central or peripheral mechanisms [1]. Central AC originates from pulmonary and cardiac disorders [1]. Peripheral AC derives either from functional vasospasm (of small cutaneous arteries, and arterioles), or compromised blood flow with stasis (in the papillary loops with aneurysmal dilatation at the tips), or higher blood viscosity due to rheological abnormalities (reduced erythrocyte flexibility, increased platelet adhesiveness, and augmented plasma viscosity factors) [1, 2, 7, 18, 35]. According to the prevailing theory, AC develops because of chronic exaggerated vasospasm of small cutaneous arteries and arterioles, blended by compensatory vasodilatation of the capillaries, and post capillary venules [1, 13, 17]. The functional alteration in vascular tone has been imputed to both genetical predisposition and autonomous (especially sympathetic hyperactivity) nervous system dysfunction [1, 35]. Fundamental vasospasm mediators comprise epinephrine, norepinephrine, serotonin, and endothelin-1 [17, 19, 20].

### 2.4. Clinical Presentation

**Figure 1:**
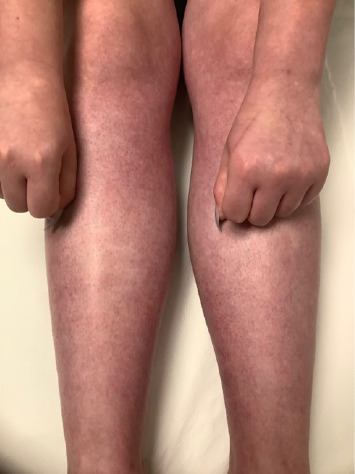
Diffuse cyanotic discoloration of the skin on the dorsa of hands, arms, and lower legs. Note the bilateral and symmetrical distribution of the discoloration and the presence of interspersed white anemic macules (Bier spots).

In primary AC, uniform violaceous to deep bluish cutaneous discoloration is usually confined to the extremities (particularly the hands and fingers; rarely the feet and toes) in a symmetrical and bilateral pattern and frequently associated with coldness and clamminess of the affected body parts [1, 11, 13, 16–19]. Extension to wrists, forearms, ankles, lower legs (Figure 1), and other acral sites (ears, nose, lips, and nipples) might be observed [1, 2, 4, 5, 8, 9, 11, 17–19]. Bier spots may be perceived as an accompanying finding (Figure 1). Palmoplantar hyperhidrosis and concomitant local edema might be detected [1, 3, 5, 9, 10, 13, 19, 36]. The dusky discoloration slowly and insidiously settles on acral skin and tends to remain constant, albeit for seasonal fluctuations in intensity. The discoloration might be aggravated during winter and alleviated during summer [1, 5, 7, 16, 17]. Cold exposure and emotional stress are likely to intensify the discoloration, while rewarming diminishes its luridness [19, 20]. The discoloration is accentuated when the extremity is lowered to a dependent position and mitigated when the extremity is positioned horizontally [9, 10, 36]. The disorder is usually asymptomatic, although paroxysmal pain may occasionally be elicited [1, 13, 18].

**Figure 2:**
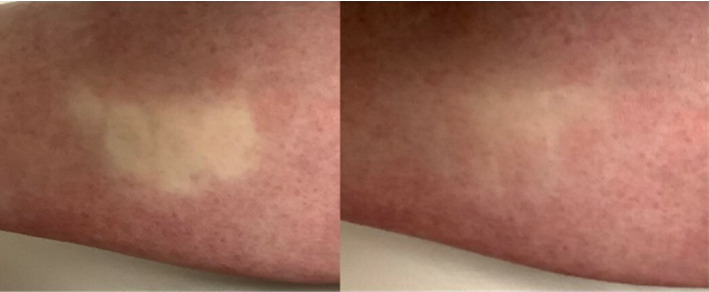
Iris shutter sign: delayed and irregular return of blood from the periphery to the center, in the white blanched area of skin created by finger pressure.

Clinically, Crocq's sign (iris shutter or iris diaphragm sign) refers to delayed and irregular blood filling from the periphery (but not from beneath) to the center, in the circumscribed blanched area of skin created by finger pressure [18] (Figure 2). Although it is a valuable clinical test, it is not pathognomonic for AC [9, 19].

**Figure 3:**
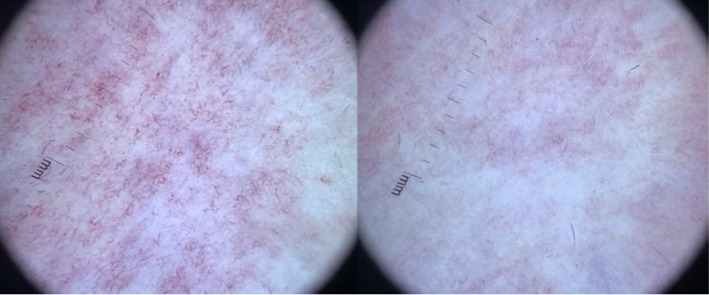
Dermatoscopy reveals prominent capillary dilatation, which becomes imperceptible when pressure is applied to the affected area with the dermatoscope.

Dermatoscopic examination in AC reveals polymorphic irregular dilated capillaries on an erythematous background, which disappear when the inspected skin area is compressed with the dermatoscope (Figure 3).

Nailfold capillary microscopy (capillaroscopy) may provide clues about microcirculation in vivo and may help in distinguishing primary AC from Raynaud's phenomenon (as the harbinger of systemic sclerosis) [37]. Nailfold capillaroscopy in AC discloses preserved capillary density, presence of microbleeds, dilated capillaries, absence of avascular zones, and presence of less than 2 giant loop capillaries (megacapillaries) per finger [37]. On the other hand, nailfold capillaroscopy in systemic sclerosis exposes low capillary density, hemorrhages, and more than 2 giant loop capillaries [37].

In contrast to primary AC, secondary AC is inclined toward an abrupt onset with asymmetrical or unilateral presentation, manifests pain, abnormal peripheral pulses, evidence of thrombosis, hypercoagulability, arterial occlusion, or DIC, elevated D-dimer levels, rapid progressive clinical course, verification of autoimmunity or multiorgan failure, association with livedo racemosa, and signs of substantial tissue damage (ulceration, necrosis, and gangrene) [5, 7, 9–11, 18, 21].

### 2.5. Diagnosis

The diagnosis of primary AC relies on typical clinical findings, and demonstration of normal results for capillary oximetry, and radioimaging studies [1, 7, 38]. Capillary oximetry typically reveals normal oxygen saturation and excludes vaso-occlusive disease [11]. Unlike secondary AC, peripheral arterial pulses are generally intact in primary AC [18, 20]. If secondary AC is suspected, appropriate laboratory and radiologic tests are performed on a case-by-case basis, to uncover the principal cause. Baseline laboratory tests such as complete blood count, ESR, CRP, LDH, CK, aldolase, nasopharyngeal swab for COVID-19, D-dimer, rheumatoid factor, antinuclear antibodies, anti-cardiolipin antibodies, serology for HBV and HCV, protein C, protein S, anti-thrombin 3, β-2 microglobulin, cryoglobulins, cryofibrinogen, and cold agglutinins may help to exclude essential thrombocythemia, vaso-occlusive disease, lymphoproliferative disease, COVID-19 infection, viral hepatitis, connective tissue disease, antiphospholipid antibody syndrome, and cold agglutinin syndrome [1, 11, 13, 19, 38]. Radioimaging (Doppler USG, CT, MRI) and nerve-conduction studies (EMG) may rule out underlying thromboembolism, neoplastic disease, spinal cord injury, and brachial plexus neuropathy [1, 11, 38]. Skin biopsy and DIF studies may assist in eliminating the prospect of cutaneous vasculitis [11].

### 2.6. Differential Diagnosis

The list of differential diagnoses includes other vascular acrosyndromes: Raynaud's disease, Raynaud's phenomenon (a paroxysmal painful condition), perniosis (a paroxysmal painful condition), acrorhigosis (subjective acral coldness without color changes), erythromelalgia (a paroxysmal painful condition), erythrocyanosis, blue finger syndrome, vaso-occlusive disease (Buerger's disease, atherosclerosis, and thromboembolic disease), BASCULE syndrome, and COVID toes/fingers [1, 7, 9, 13, 19]. Concentrating merely on cold-induced/cold-exacerbated primary acral vasospastic disorders, the differential diagnosis might be constrained to a few in number (Table 2). Nonetheless, it is important to keep in mind that AC may overlap/coexist with other acrosyndromes (perniosis, erythromelalgia, and Raynaud's disease) and livedo reticularis [3, 9, 18, 19, 21, 39–41].

AC is arbitrarily differentiated from Raynaud's disease by relative persistence of skin discoloration in warm and cold weather, absence of marked symptomatology, and lack of paroxysmal color changes in the former [9, 13, 19, 20]. AC does not display the episodic triphasic (pallor, cyanosis, rubor) or biphasic (pallor, cyanosis) color changes archetypal of Raynaud's disease [1, 6, 16, 19]. Of note, Raynaud's disease preferentially involves the fingers, and toes, in short-lived, painful attacks of 15–20-minute duration [3, 8, 18, 36].

Pernio (chilblains) is an acral, cold-induced, episodic, and reversible inflammatory disorder, characterized by localized, circumscribed, discrete, erythematous, purplish, and edematous macules, papules, nodules, and plaques [18, 19, 36]. Pernio lesions spout usually on the dorsa/pads of toes and fingers with an abrupt onset, elicit itching, tenderness, burning, or pain sensations, and usually resolve within 3 weeks [9, 18, 19, 21, 36].

Erythromelalgia (erythermalgia) is a rare vascular acrosyndrome, characterized by the triad of erythema, warmth, and burning pain, usually involving the lower limbs, feet, and toes, symmetrically and bilaterally [18, 40, 51]. Paradoxically, the symptomatic episodes, which may continue from minutes to days, are precipitated by heat and relieved by cold [51]. However, between the episodes, patients with erythromelalgia may have cool, discolored extremities simulating AC or Raynaud's disease [9, 41]. Although it is not a cold-induced vasospastic acrosyndrome, complications (infection, ischemia, ulcerations, necrosis, and gangrene) might ultimately arise because of excessive cold exposure by patients to relieve the symptoms [51].

Erythrocyanosis refers to a cyanotic discoloration over skin areas with a thick layer of subcutaneous fat [52].

Livedo reticularis is a cold-induced, asymptomatic, symmetrical, and reversible acral ischemic disorder, characterized by violaceous uniform reticular or fishnet-like mottling surrounding pale skin [18, 19]. Livedo racemosa, the pathologic, secondary, and persistent variant of livedo reticularis, may have irregular, and asymmetrical distribution, and may elicit pain sensation [18, 19]. In contrast, primary or isolated AC does not display a reticular pattern.

AC is one of the initial manifestations of systemic sclerosis and appears years before Raynaud's phenomenon and actual sclerosis supervene [22]. Vascular occlusion or peripheral vasospasm in systemic sclerosis is evident through several acral vasculopathic abnormalities, i.e., AC, Raynaud's phenomenon, telangiectasias, digital ulcers, gangrene, and peripheral pulse deficiency [22].

Dependent AC is a well-known dermatologic manifestation encountered in almost half of patients with postural orthostatic tachycardia syndrome (POTS) [23].

AC is also a component of BASCULE (Bier anemic spots, cyanosis, and urticaria-like eruption) syndrome, a primary, though episodic, vasomotor entity, that occurs in the setting of POTS and long COVID-19 syndrome [24–26]. Clinically, it is characterized by Bier spots, erythrocyanosis, and urticarial plaques located on the lower limbs, which appear in dependent position and disappear with limb movement [24, 26, 27]. When visible, the skin lesions are associated with tenderness, pruritus, and sometimes pain sensations [24].

Bier spots (angiospastic macules and physiologic anemic macules) are characterized by transient, benign, multiple, pale to ivory-white, 1–10 mm macules on a background of diffuse erythema or cyanosis, symmetrically distributed over the extensor surface of distal limbs [53]. The lesions are more noticeable in dependent position and vanish upon limb elevation [53]. Physiologically, they develop because of focal exaggerated vasospasm of small blood vessels [53]. Bier spots may be observed as an isolated phenomenon or accompany BASCULE and other acrosyndromes [53].

Along with the coronavirus pandemic, a sudden outburst of skin signs resembling vascular acrosyndromes was observed by dermatologists [15, 28]. The potpourri of chilblain-like (perniosis-like) acral lesions (CLL) and/or AC in the setting of COVID-19 infection has been termed COVID toes/fingers [19, 28, 42–44]. Although the exact prevalence remains unknown, thousands of CLL cases coupled to or followed by AC have been reported worldwide during the COVID-19 lockdowns [14, 15, 45]. Subsequently, mRNA-based COVID-19 vaccines have also been incriminated in the development of CLL and AC (vaccine toes) [46, 47].

CLL remains as the most common cutaneous manifestation liable to COVID-19 infection [28, 38, 42–44]. It is believed to signify an innate immune reaction to SARS-CoV-2 spike protein, through type I IFN secretion [14, 28, 42, 44, 46]. It is usually a late manifestation and generally confronted in children and young adults with milder or asymptomatic COVID-19 infection, foretelling a good prognosis [28, 38, 43, 46, 48]. The clinical picture is reminiscent of classical chilblains (perniosis); however, attacks of CLL occur not only during cold climates, but also in warm, and even hot weather [14, 38]. Unlike AC, CLL is recognized by its abrupt onset, predominant involvement of the toes, circumscription of the erythematous, edematous, bullous, or purpuric lesions, presence of itching, pain, and tenderness sensations, and favorable outcome with spontaneous resolution within 3 months, albeit with a recurrent course [13, 14, 43, 48]. Once again, caution is required, as CLL may be blended with AC and livedo reticularis in the late course of the long COVID-19 syndrome [39, 45].

With the flood of publications, both COVID-19 and novel mRNA-based vaccines for COVID-19 have been affixed to the list of disorders prompting secondary AC [29, 30, 47]. COVID-19 infection/vaccination may not only provoke acral vasospastic but also acral ischemic AC-like portraits (COVID foot). This latter clinical picture, attributed to thrombotic coagulopathy and predicting a poor prognosis, is encountered in adults with underlying severe systemic SARS-CoV-2 infection or long COVID-19 syndrome, or arises from abnormal exaggerated immune response to COVID-19 vaccination [19, 25, 28, 42, 47–50].

### 2.7. Treatment

Unfortunately, effective medical therapy is lacking in primary AC and supportive care is the mainstay of treatment [1, 9, 12]. Treatment is directed to the underlying disorder in secondary AC [9, 11, 13]. In mild primary AC, lifestyle modification (limiting cold exposure, cessation of smoking, limiting the intake of caffeine, and wearing of protective garments like gloves, socks, slippers, insulated clothing, and boots), dietary and hygiene counseling, avoidance of trauma, gentle warming and massage of the affected areas, and reassurance on the benign nature of the disorder may be all that is required [1, 5]. There is neither consensus nor evidence-based guidelines/algorithms for therapy in severe, recurrent, or persistent AC. Although none of them has been approved hitherto, vasodilators (calcium channel blockers (nifedipine, verapamil, or diltiazem) and alpha-adrenergic blockers (prazosin and anisodamine)), anti-platelet drugs (aspirin/ticlopidine and clopidogrel), rheological agents that improve peripheral blood flow (pentoxifylline and cilostazol), prostaglandin analogs (iloprost and misoprostol), ketanserin (a selective serotonin antagonist), topical nicotinic acid derivatives (such as hexyl nicotinate 2% cream), topical nitroglycerine, topical salbutamol sulfate, topical minoxidil, and ultraviolet light (UVB) have been used for treating AC [1, 6, 7, 9, 11, 16, 19, 31, 52]. Even though the archaic English literature cites β-blockers as therapeutic alternatives in AC, they may paradoxically aggravate AC through vasoconstrictive effects, and as such, remain contraindicated [32]. Sympathetic nerve block and sympathectomy are occasionally attempted in severe, recurrent, or persistent AC cases [1, 9].

### 2.8. Prognosis

Primary AC is usually a benign, but cosmetically disfiguring disorder [1, 11]. It typically resolves spontaneously by middle adulthood and does not pose a risk of short or long-term sequelae [9, 16]. Therefore, it has a favorable prognosis as compared with other acrosyndromes [5, 13]. Secondary AC has a vague prognosis that depends on the underlying cause and correlates with its severity, and as such, it may portend a poorer prognosis compared to primary AC [9, 11].

## 3. Discussion/Results

This is a concise narrative review with the intention to reflect a dermatologic perspective on AC in the light of the COVID-19 pandemic. There is paucity of randomized, double-blind, placebo-controlled studies on AC in the English literature, implicating the need for targeted research. Pertinent information on AC still largely relies on anecdotal observations, case reports, case series, or scant reviews, which are dated rather old and published in vascular-oriented journals. The scarcity of published literature on AC and other acrosyndromes in dermatology-oriented journals points to the necessity of additional professional education and further improvement of clinical diagnostic skills for dermatologists.

Despite an increasing prevalence and a boost of publications during the COVID-19 outbreak, AC is still misdiagnosed, underdiagnosed, and underreported by the dermatology community. This may be attributed both to the presence of substantial overlap among acrosyndromes, and to lack of knowledge and/or interest in cutaneous vascular medicine. However, once the clinical cutaneous portrait is realized, a confident straightforward diagnosis of AC is not challenging for a dermatologist. Rather, because of its complex etiopathogenesis, unearthing the underlying grounds in secondary AC appears to be a more difficult task.

## 4. Conclusions

Although it has been described for over a century ago, AC remains as the least recognized and the least studied acrosyndrome. Future research should address the existing gaps in AC epidemiology, etiology, pathophysiology, and treatment. The most relevant causes of secondary AC demand to be explored. The hormonal aspect of acrosyndromes deserves further appraisal. Therapeutic agents that block the peripheral effects of vasospasm mediators, either in topical or oral formulations, warrant testing in prospective clinical trials. By extrapolation from preexisting data, therapeutics that have shown benefit in BASCULE syndrome (high-dose antihistamines) [27] may also be tried in patients with AC. Whether acrosyndromes are distinct dermatoses or reside on a continuous spectrum remains to be determined. Randomized, double-blind, placebo-controlled studies might be conducted by multidisciplinary and international collaboration. Dermatologists should be encouraged to publish more and take an active role in targeted research, diagnosis, and management of AC and other acrosyndromes.

## Figures and Tables

**Table 1:** Disorders reported in association with or underlying acrocyanosis [2, 4–11, 18–34].

Psychiatric and nutritional	Bipolar disorder, Asperger's syndrome, schizophrenia, mental retardation, chronic starvation, anorexia nervosa, bulimia nervosa
Coronary, vascular, and orthostatic	Hypoxemia with peripheral cyanosis, high altitude, stroke, myocardial infarction, arterial occlusive disease (atheromatous/septic embolism), coagulopathy (disseminated intravascular coagulation, heparin-induced thrombocytopenia), Buerger's disease, thromboembolic disease, postural orthostatic tachycardia syndrome (POTS), chronic orthostatic intolerance, BASCULE syndrome, chronic fatigue syndrome, hypersensitivity coronary syndrome (Kounis syndrome), vasculitis, arteritis
Pulmonary	Pulmonary hypertension, pulmonary embolism, pulmonary alveolar proteinosis, pulmonary arteriovenous malformations, acrocyanosis of infancy, atheromatous embolism
Hematologic and rheologic	Essential thrombocythemia, paroxysmal cold hemoglobinuria, cryoglobulinemia, cryofibrinogenemia, cold agglutinin syndrome (primary or secondary to *M. pneumoniae* infection, infectious mononucleosis, CMV, mumps, influenza, congenital and acquired syphilis, varicella, rubella, parvovirus B19, *Chlamydia psittaci*, *Legionella*, *Citrobacter*, *Listeria monocytogenes*)
Autoimmune	Systemic lupus erythematosus, scleroderma (systemic sclerosis), rheumatoid arthritis, dermatomyositis, mixed connective tissue disease, primary and secondary antiphospholipid antibody (APLA) syndrome, Wegener's granulomatosis, overlap syndrome
Neoplastic and paraneoplastic	Lymphoproliferative disorders, myeloproliferative disorders, benign and malignant paraproteinemia, POEMS syndrome, multicentric Castleman's disease, ovarian cancer, Hodgkin's lymphoma, chronic lymphocytic leukemia
Neurologic and traumatic	Spinal cord injury, peripheral neuropathy, multiple sclerosis, brachial plexus neuropathy, cervical plexus compressive neuropathy (supernumerary cervical rib, thoracic outlet syndrome, Klippel–Feil syndrome, scalenus anterior syndrome), carpal tunnel syndrome, vegetative dystonia, neurocirculatory asthenia, familial spastic paraplegia
Therapeutic and toxic	Interferon alfa 2a, interferon beta, β-blockers, cocaine, ergotamine, nicotine, caffeine, oral contraceptives, HRT, tricyclic antidepressants (imipramine, desipramine), selective serotonin reuptake inhibitors (fluoxetine), vasopressors (terlipressin, dopamine), butyl nitrate, valproic acid, sirolimus, norepinephrine, phenylephrine, pseudoephedrine, clonidine, alphaprodine, amphotericin B, phenazopyridine, benzocaine, propoxyphene, gemcitabine, cisplatin, oxaliplatin, bleomycin, vincristine, IVIG, mercury poisoning (acrodynia), arsenic poisoning (black foot disease), ergotism, blasticidin-S, metoclopramide, dapsone, diclofenac, immune checkpoint inhibitors (ipilimumab + nivolumab, pembrolizumab, tremelimumab + durvalumab), natalizumab, mRNA-based COVID-19 vaccines
Metabolic and genetic	Fucosidosis, ethyl malonic aciduria, cytochrome C oxidase deficiency, hyperoxaluria type I, mitochondrial disease (oxidative phosphorylation disorders), spondyloenchondrodysplasia, palmoplantar keratoderma, Down's syndrome, Prader–Willi syndrome, Sneddon's syndrome, Aicardi–Goutieres syndrome, Marfan's disease, Riley–Day syndrome (familial dysautonomia), Ehlers–Danlos syndrome
Infectious	HIV, psittacosis, chikungunya, HCV, parvovirus B19, *M. leprae*, *B. burgdorferi*, COVID-19 and long COVID-19 syndrome
Occupational	Working with vibratory tools, exposure to solvents
Allergic and miscellaneous	Puffy hand syndrome (hand sclerosis and lymphedema due to narcotic injections, often associated with HCV infection and rarely with pregnancy), Pickwickian syndrome, ozena, chronic hypertrophic and primary atrophic rhinitis, atopic dermatitis, frostbite

**Table 2:** Clinical comparison of cold-induced or cold-exacerbated, primary acral vasospastic disorders [1, 3, 6–9, 11, 13–16, 18–21, 28–30, 36, 39, 42–50].

	Acrocyanosis	Raynaud's disease	Pernio (chilblains)	Livedo reticularis	COVID toes/fingers
Gender and age	Females; before 25–30 years	Females; before 40 years	Females; young, middle-aged	Females; 20–60 years	Any gender; CLL in children, adolescents, and young adults; acroischemic lesions in adults and elderly
Triggering/exacerbating factor	Cold, emotional stress	Cold, emotional stress	Cold, damp	Cold	Cold, damp, warm, heat
Onset	Gradual	Abrupt	Abrupt	Abrupt	Abrupt
Duration	Persistent, despite seasonal fluctuations in intensity	Episodic; 15–20 min	Episodic; a few days to 3 weeks	Episodic	Episodic; a few days to 3 months, rarely longer
Morphology of lesions	Uniform cyanotic discoloration	Triphasic/biphasic color change	Discrete and circumscribed, red-purple edematous papules, nodules, plaques, ulceration, necrosis	Uniform violaceous reticular, or fishnet-like mottling	Pernio-like (CLL) or AC-like morphology
Predominant location of lesions	Hands > feet and digits (fingers > toes)	Digits (fingers > toes)	Dorsa and pads of toes > fingers	Extremities (lower > upper)	Dorsa and sides of toes > fingers, and feet > hands
Distribution of lesions	Symmetrical, bilateral	Symmetrical, bilateral	Depends on exposure	Symmetrical, bilateral	Symmetrical, bilateral, rarely asymmetrical
Symptomatology	Asymptomatic	Pain, paresthesia	Pain, tenderness, burning, itching	Asymptomatic	Pain, tenderness, itching

## Data Availability

Data sharing is not applicable to this article as no datasets were generated or analyzed during the current study.

## References

[1] Das S., Maiti A. (2013). Acrocyanosis: An Overview. *Indian Journal of Dermatology*.

[2] Glazer E., Pacanowski J. P., Leon L. R. (2011). Asymptomatic Lower Extremity Acrocyanosis: Report of Two Cases and Review of the Literature. *Vascular*.

[3] Fontaine C., Staumont-Sallé D., Hatron P. Y., Cotten A., Couturier C. (2014). The Hand in Systemic Diseases Other Than Rheumatoid Arthritis. *Chirurgie de la Main*.

[4] Mosdósi B., Nyul Z., Nagy A., Bölcskei K., Decsi T., Helyes Z. (2017). Severe Acrocyanosis Precipitated by Cold Agglutinin Secondary to Infection With Mycoplasma Pneumoniae in a Pediatric Patient. *Croatian Medical Journal*.

[5] Putz A., Metz S., Elsner P. (2014). Nonspecific Unilateral, Painful Acrocyanosis--A Dermatological Case? Symptomatic Stenosis of the Axillary Artery. *JDDG: Journal der Deutschen Dermatologischen Gesellschaft*.

[6] Kumar K., Kumar A., Tomar D., Gupta A. K. (2014). Acrocyanosis With Intrahepatic Carcinoid Tumor. *Indian Dermatology Online Journal*.

[7] Takeuchi Y., Tsukagoshi J. (2021). Primary Acrocyanosis. *Journal of General and Family Medicine*.

[8] Wollina U., Koch A., Langner D. (2018). Acrocyanosis - A Symptom With Many Facettes. *Open Access Macedonian Journal of Medical Sciences*.

[9] Kurklinsky A. K., Miller V. M., Rooke T. W. (2011). Acrocyanosis: The Flying Dutchman. *Vascular Medicine*.

[10] Ruiz-Rodríguez J. F., Fernández-de Thomas R. J., De Jesus O. (2022). Secondary Acrocyanosis in a Paraplegic Patient With Spinal Cord Injury. *Cureus*.

[11] Zakaria E. R., Wan Ghazali W. S., Sazali H., Shahril N. S., Md Salleh S., Mohd Nawi S. N. (2024). Acrocyanosis as a Rare Presentation of Drug-Induced Cutaneous Vasculitis: A Case Report. *BMC Rheumatol.*.

[12] Heidrich H. (2010). Functional Vascular Diseases: Raynaud’s Syndrome, Acrocyanosis and Erythromelalgia. *Vasa*.

[13] Kent J. T., Carr D. (2021). A Visually Striking Case of Primary Acrocyanosis: A Rare Cause of the Blue Digit. *The American Journal of Emergency Medicine*.

[14] Colonna C., Restano L., Monzani N. A. (2022). Rare and Common Manifestations of COVID-19 in Children. *JEADV Clinical Practice*.

[15] Tosti G., Barisani A., Queirolo P. (2020). Skin Signs Resembling Vascular Acrosyndromes During the COVID-19 Outbreak in Italy. *Clinical and Experimental Dermatology*.

[16] Das S., Roy A. K., Maiti A. (2010). Remittent Idiopathic Necrotizing Acrocyanosis - A Rare Entity. *Indian Journal of Dermatology*.

[17] Kumar P., Ghosh S., Tanwar H. S., Gupta A. K. (2014). Acrocyanosis in a Young Adult: A Rare Presentation of Extra-Adrenal Pheochromocytoma. *BMJ Case Reports*.

[18] Dean S. M. (2018). Cutaneous Manifestations of Chronic Vascular Disease. *Progress in Cardiovascular Diseases*.

[19] Choi E., Henkin S. (2021). Raynaud’s Phenomenon and Related Vasospastic Disorders. *Vascular Medicine*.

[20] Nousari H. C., Kimyai-Asadi A., Anhalt G. J. (2001). Chronic Idiopathic Acrocyanosis. *Journal of the American Academy of Dermatology*.

[21] Middleton H. T., Boswell C. L., Houwink E. J., Allen-Rhoades W. A., Kuhn A. K., Wright J. A. (2023). Vincristine-Induced Acrocyanosis and Erythema Pernio. *J Prim Care Community Health*.

[22] Bobeica C., Niculet E., Musat C. L. (2024). The Association of Telangiectasias With Other Peripheral Vascular Lesions of Systemic Sclerosis. *Clinical, Cosmetic and Investigational Dermatology*.

[23] Kaur N., Arun V. A., Rana S., Sashindran V. K. (2017). Benign Joint Hypermobility Syndrome With Postural Orthostatic Tachycardia Syndrome and acrocyanosisJoint Hypermobility Syndrome and Dysautonomia. *Medical Journal of Dr. D.Y. Patil University*.

[24] Baurens N., Briand C., Giovannini-Chami L. (2022). Case Report, Practices Survey and Literature Review of an Under-Recognized Pediatric Vascular Disorder: The BASCULE Syndrome. *Frontiers in Pediatrics*.

[25] Iftekhar N., Sivan M. (2023). Venous Insufficiency and Acrocyanosis in Long COVID: Dysautonomia. *The Lancet*.

[26] Danescu S., Baican C., Chiorean R., Filip M., Cismaru G., Baican A. (2018). Orthostatic Hypotension Revealed by BASCULE Syndrome. *European Journal of Dermatology*.

[27] Reinhart J. P., Kumar A. B., Casanegra A. I. (2024). Bridging the Gap in BASCULE Syndrome: A Retrospective Case Series of a Recently Described Clinical Entity. *Pediatric Dermatology*.

[28] Demircioğlu D., Durmaz E. Ö, Şahin S. (2023). Cyanotic Fingers and Omicron, Oh My. *Indian Journal of Dermatology*.

[29] Agarwal A., Sharma A., Jakhar R., Ag M. (2021). Severe Acute Acrocyanosis and Digital Gangrene as a Sign of Catastrophic COVID-19 Infection. *Journal of Clinical and Diagnostic Research*.

[30] Wang H. J., Sun Y., Lan C. E. (2022). Systemic Lupus Erythematosus With Acrocyanosis After AstraZeneca COVID-19 Vaccination. *The Kaohsiung Journal of Medical Sciences*.

[31] Reijers I. L. M., Hilhorst M., Tan M., Klarenbeek P. L., Hak A. E., Blank C. U. (2020). Acrocyanosis after Neoadjuvant Ipilimumab Plus Nivolumab: A Case Report. *Clinical & Experimental Rheumatology*.

[32] Tatu A. L., Elisei A. M., Chioncel V., Miulescu M., Nwabudike L. C. (2019). Immunologic Adverse Reactions of β-Blockers and the Skin. *Experimental and Therapeutic Medicine*.

[33] Cohen P. R. (2024). Injected Drug Addiction-Associated Swollen Hands: A Case Report of Methylamphetamine-Related Unilateral Drug Addiction-Related Puffy Hand Syndrome. *Cureus*.

[34] Khaddour K., Singh V., Shayuk M. (2019). Acral Vascular Necrosis Associated With Immune-Check Point Inhibitors: Case Report With Literature Review. *BMC Cancer*.

[35] Ohtake N., Sou K., Tsukamoto K., Furue M., Tamaki K. (1995). Diffuse Palmoplantar Keratoderma Associated With Acrocyanosis and Livedo Reticularis. Two Sporadic Cases. *Acta Dermato-Venereologica*.

[36] Bergersen T. K., Walløe L. (2018). Acral Coldness - Severely Reduced Blood Flow to Fingers and Toes. *Handbook of Clinical Neurology*.

[37] Guelimi R., Monfort J. B., Chaby G. (2024). Nailfold Capillaroscopy in Acrocyanosis Among Patients With Associated Raynaud’s Phenomenon. *Annales de Dermatologie et de Vénéréologie*.

[38] Sachdeva M., Mufti A., Maliyar K., Lara-Corrales I., Salcido R., Sibbald C. (2021). A Review of COVID-19 Chilblains-Like Lesions and Their Differential Diagnoses. *Advances in Skin & Wound Care*.

[39] García-Gil M. F., Monte Serrano J., Lapeña-Casado A., García García M., Matovelle Ochoa C., Ara-Martín M. (2020). Livedo Reticularis and Acrocyanosis as Late Manifestations of COVID-19 in Two Cases with Familial Aggregation. Potential Pathogenic Role of Complement (C4c). *International Journal of Dermatology*.

[40] Leroux M. B. (2018). Erythromelalgia: A Cutaneous Manifestation of Neuropathy?. *Anais Brasileiros de Dermatologia*.

[41] Davis M. D., Wilkins F., Rooke T. W. (2006). Between Episodes of Erythromelalgia: A Spectrum of Colors. *Archives of Dermatology*.

[42] Panda M., Agarwal A., Hassanandani T. (2022). Dermatological Manifestations of COVID-19 in Children. *Indian Pediatrics*.

[43] Lee S. S., Mancuso J., Tracy A., Eichenfield L. F. (2021). Not COVID Toes: Pool Palms and Feet in Pediatric Patients. *Cutis*.

[44] Huynh T., Sanchez-Flores X., Yau J., Huang J. T. (2022). Cutaneous Manifestations of SARS-CoV-2 Infection. *American Journal of Clinical Dermatology*.

[45] Ganatra B., Amarnani R., Alfallouji Y. (2022). Patient Characteristics in Tardive COVID-19 Pseudoperniosis: A Case Series of 16 Patients. *Clinical and Experimental Dermatology*.

[46] Hodge B., Daniel C. R., Elewski B. E. (2022). Vaccine Toes Are the New COVID Toes. *Skin Appendage Disorders*.

[47] Cavazos A., Deb A., Sharma U., Nugent K. (2022). COVID Toes Following Vaccination. *Baylor University Medical Center Proceedings*.

[48] Kashetsky N., Mukovozov I. M., Bergman J. (2021). Chilblain-Like Lesions (CLL) Associated With COVID-19 (“COVID Toes”): A Systematic Review. *Journal of Cutaneous Medicine and Surgery*.

[49] Pourdowlat G., Naderi Z., Seif F., Mansouri D., Raji H. (2020). Acrocyanosis and Digital Necrosis Are Associated With Poor Prognosis in COVID-19. *Clinical Case Reports*.

[50] Kolivras A., Thompson C., Pastushenko I. (2022). A Clinicopathological Description of COVID-19-Induced Chilblains (COVID-Toes) Correlated With a Published Literature Review. *Journal of Cutaneous Pathology*.

[51] Tham S. W., Giles M. (2018). Current Pain Management Strategies for Patients with Erythromelalgia: A Critical Review. *Journal of Pain Research*.

[52] Singh G. (2017). High Altitude Dermatology. *Indian Journal of Dermatology*.

[53] Demirciogˇlu D., Durmaz E. Ö (2022). Polka Dot Appearance on the Face. *JDDG: Journal der Deutschen Dermatologischen Gesellschaft*.

